# Investigation of structural, optical and electrical conductivity of a new organic inorganic bromide: [C_12_H_17_N_2_]_2_ZnBr_4_†

**DOI:** 10.1039/d3ra00561e

**Published:** 2023-03-10

**Authors:** I. Kammoun, M. Belhouchet, A. Ben Ahmed, J. Lhoste, M. Gargouri

**Affiliations:** a University of Sfax, Faculty of Sciences, Laboratory of Spectroscopic Characterization and Optical Materials 3018 Sfax BP1171 Tunisia kammoun.fss@gmail.com; b Physico-Chemistry of Solid State Laboratory, Department of Chemistry, Faculty of Sciences of Sfax 3000 Sfax BP1171 Tunisia; c University of Sfax, Faculty of Sciences of Sfax, Department of Physic, Laboratory of Applied Physic B.P. No. 802 3018 Sfax Tunisia; d Institute for Molecules and Materials Le Mans, University of Maine Avenue Olivier Messiaen 72085 Le Mans Cedex 9 France

## Abstract

A new organic–inorganic hybrid, namely the [C_12_H_17_N_2_]_2_ZnBr_4_ compound, has been synthesized and studied by single-crystal X-ray diffraction and optical and complex impedance spectroscopy. It crystallized in the centrosymmetric *P*2_1_/*n* space group at room temperature. The asymmetric unit is constituted by [ZnBr_4_]^2−^ anions, showing slightly distorted tetrahedral geometry, surrounded by four organic (C_12_H_17_N_2_)^+^ cations. The crystal packing is stabilized by N–H⋯Br and C–H⋯Br hydrogen bonds arranged in a three-dimensional network. The optical absorption measurement confirms the semiconductor nature with a band gap of around 3.94 eV. Additionally, the analysis of Nyquist plots (−*Z*′′ *vs. Z*′) shows that the electrical properties of the material are heavily dependent on frequency and temperature, indicating a relaxation phenomenon and semiconductor-type behavior. Reduction in *Z*′ was observed as a function of temperature and frequency which indicates an increase in ac conductivity and the negative temperature coefficient of resistance. The frequency dependent plots of (−Z′′) show that the electrical relaxation is non-Debye in nature. The ac conductivity spectrum obeys Jonscher's universal power law. The Correlated barrier hopping model CBH has been suggested to agree with the conduction mechanism of *σ*_*ac*_ for the [C_12_H_17_N_2_]_2_ZnBr_4_ compound.

## Abstract 2

A new organic–inorganic hybrid, namely the [C_12_H_17_N_2_]_2_ZnBr_4_ compound, has been synthesized and studied by single-crystal X-ray diffraction and optical and complex impedance spectroscopy.

## Graphical abstract

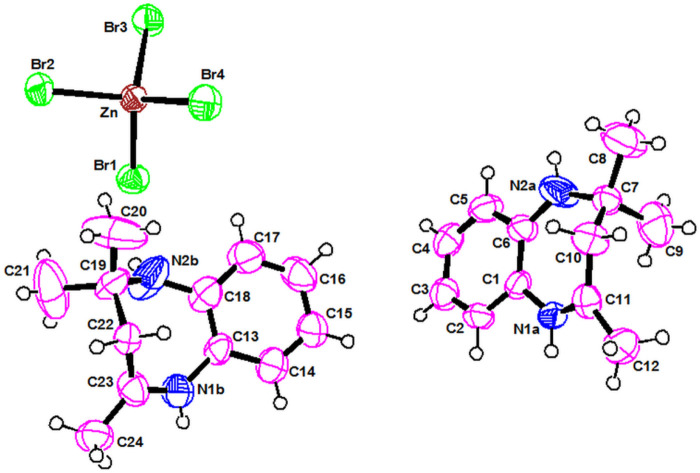

## 1. Introduction

In recent years, organic–inorganic hybrid compounds have constituted a large class of materials thanks to the improvement of their physico-chemical properties through which it was possible to gather both organic and inorganic components as well as outstanding properties from the different parts.^1–3^ A special place in this group of materials is occupied by organic–inorganic hybrids based on divalent metal halides (Cu, Zn, Co, Ni…) whose temperature-dependent optical and electrical properties are relevant for emerging applications in optical devices, information storage, solar cells, photocatalysts, *etc.*^4–8^ An important feature of organic–inorganic hybrids is their structural tenability. In fact, some structural differences are observed if the counter ions of the inorganic parts are monoprotonated or diprotonated amines. The crystallographic orientation and the thickness of the inorganic sheets may vary according to the choice of the appropriate organic cations.^9,10^ Due to the structural flexibility of the hybrid compound, the choice of different organo-ammoniums to stabilize various orientations or dimensions of inorganic sheets is an interesting area to explore the relationship between structures and physico-chemical properties within a single structure family.^11,12^ The organic divalent zinc bromide has several applications in various domains such as catalysis, biochemistry, dielectric transition and magnetism material science. In addition, the study of such zinc-based materials has received much attention recently in the light of photovoltaic and multifunctional properties.^13^

The new [C_12_H_17_N_2_]_2_ZnBr_4_ compound has therefore been prepared with the aim of studying its optical and electrical properties by using UV-vis and complex impedance spectroscopy, respectively. Its crystal structure was determined from X-ray diffraction data collected on a single crystal obtained by the slow evaporation method.

## 2. Experimental

### 2.1. Synthesis

The synthesis of the [C_12_H_17_N_2_]_2_ZnBr_4_ sample was carried out using the reported preparation procedure.^14^ The organic parts 1,2-phenylenediamine (purity 99.5%, FLUKA) was dissolved in acetone and ZnBr_2_ (purity 98%, FLUKA), dissolved in hydrochloric acid solution (1 M), in molar ratio 2 : 1. By slow evaporation at room temperature, yellow crystals suitable for X-ray single crystal analysis were obtained.

### 2.2. X-ray data collection

Single crystal sized (0.35, 0.25, 0.20) mm^3^ was carefully selected to perform its structural analysis by X-ray diffraction. The crystallographic data were collected on a Bruker AXS CCD diffractometer at room temperature using graphite-monochromated Mo Kα radiation (*λ* = 0.71073 Å). All intensities were corrected for Lorentz, polarization and absorption effects.^15^ The structural determination procedure was carried out using SHELXS97 program.^16^ The structure was solved by direct method and refined with full-matrix least squares methods based on *F*^2^ using SHELXL97.^17^ The space group was determined to be *P*2_1_/*n*. A total of 54568 reflections were collected in the *θ* range 2.2–27.5°. In this structure, all non-hydrogen atoms were refined with anisotropic displacement parameters. H-atoms were set in calculated positions and treated as riding on their parent atom with constrained thermal parameters. The final discrepancy factors *R*_1_ and w*R*_2_ are 0.053 and 0.134, respectively. Crystal data of (C_12_H_17_N_2_)_2_ZnBr_4_ are given in Table 1. Molecular plots were made with ORTEP^18^ and Diamond.^19^

**Table 1:** Crystal data and structure refinement of [C_12_H_17_N_2_]_2_[ZnBr_4_]

Empirical formula	[C_12_H_17_N_2_]_2_[ZnBr_4_]
Formula weight	763.56
Temperature (K)	296
Wavelength (Å)	0.71073
Crystal system, space group	Monoclinic, *P*2_1_/*n*
Unit cell dimensions	
*a* (Å)	12.1363(6)
*b* (Å)	14.9001(8)
*c* (Å)	16.2525(9)
*β* (°)	98.612(2)
*V* (Å^3^)	2905.8(3)
*Z*	4
Density (calculated) (g cm^−3^)	1.745
Reflections collected	54568
Independent reflections	6663
Reflections observed with *I* > 2*σ*(*I*)	3046
*R*_int_	0.136
Number of refined parameters	298
Goodness-of-fit on *F*^2^	0.99
Final *R* indices [*I* > 2*σ*(*I*)]	*R*_1_ = 0.053 and w*R*_2_ = 0.134
Largest diff. peak and hole, *e* Å^−3^	1.02 and −0.90
CCDC no.	2 090 035

Atomic coordinates anisotropic displacement parameters, tables for all bond distances, and angles have been deposited at the Cambridge Crystallographic Data Centre (deposition number: CCDC 2090035).

### 2.3. Impedance spectroscopy

Furthermore, the impedance measurements of the investigated [C_12_H_17_N_2_]_2_ZnBr_4_ compound were performed using two platinum electrodes. As a fact, the fine grain samples were pressed into a cylindrical pellet of typical dimension 8 mm diameter and 1.1 mm thickness pressed at a pressure of 3 ton per cm^2^. Then, thin gold films, (with a thickness of a few nanometers), were manually deposited on both flat faces of the pellet. The measurements were performed as a function of both temperature and frequency employing using an 1260 Solartron Impedance Analyzer.

### 2.4. UV-visible measurements

UV-visible measurement was carried out using a conventional UV-visible spectrophotometer (HITACHI, U-3300) in the range 200–800 nm.

## 3. Results and discussion

### 3.1. Crystal structure

**Fig. 1:**
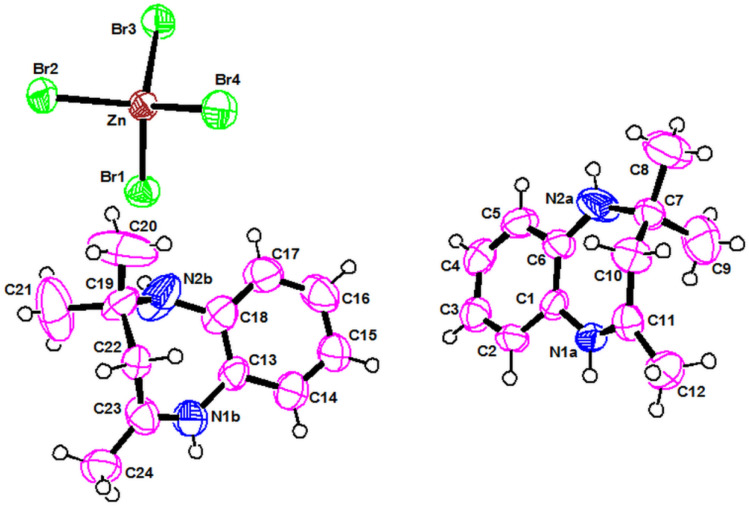
Molecular structure of the asymmetric unit of [C_12_H_17_N_2_]_2_[ZnBr_4_].

As shown in Fig. 1, the asymmetric unit is constituted by one tetabromozincate [ZnBr_4_]^2−^ anion and two protonated [C_12_H_17_N_2_]^+^ cations. The zinc atom is coordinated to four Br atoms forming a slightly distorted tetrahedron, as it can be deduced from the value of both the Zn–Br bond lengths, ranging from 2.3638(11) to 2.4464(10) Å, and the Br–Zn–Br angles, ranging from 105.35(4)° to 113.89(4)° (Table S1)†. The structural parameters of the present compound agree well with those found in similar compounds characterized by [ZnBr_4_]^2−^ tetrahedral units.^20^

The two chemically identical monoprotonated cations [C_12_H_17_N_2_]^+^ are crystallographically independent and are noted respectively: Cation (A) {N1a, N2a} and Cation (B) {N2a, N2b}. The values of C–C and C–N distance, in the two cations, range from 1.284(8) to 1.539(10) Å while C–C–C and C–C–N angles are between 105.2(7) and 133.3(6) (Table S2)†. Both cations are ordered and have slightly different torsions angles.^21,22^

**Fig. 2:**
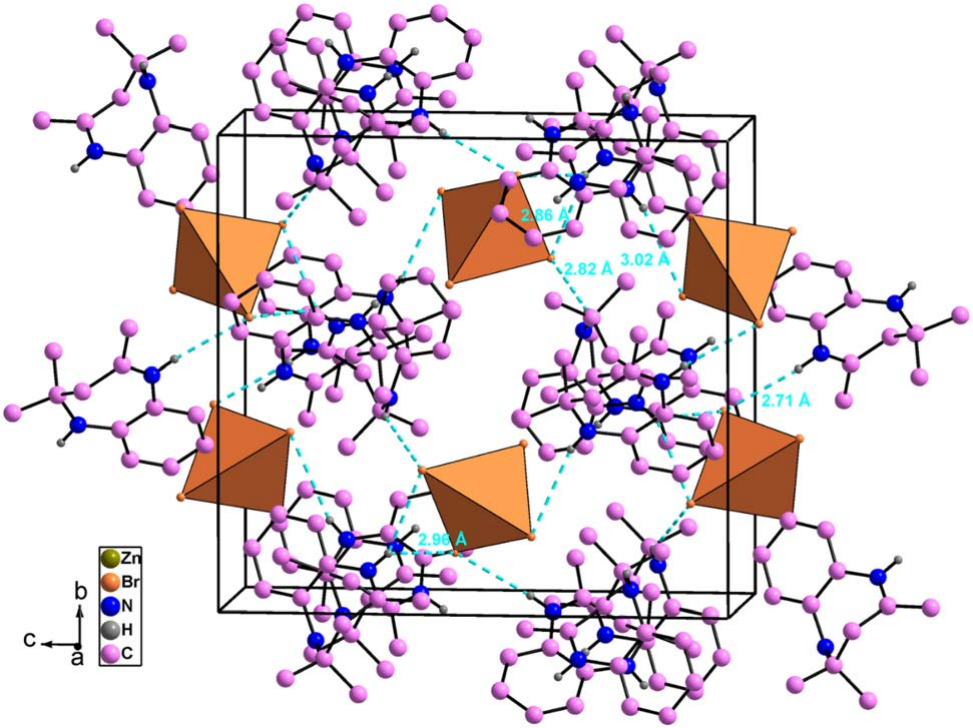
Crystal packing of [C_12_H_17_N_2_]_2_[ZnBr_4_] compound in the (*b*, *c*) plane (hydrogen bonds shown as dashed lines).

**Fig. 3:**
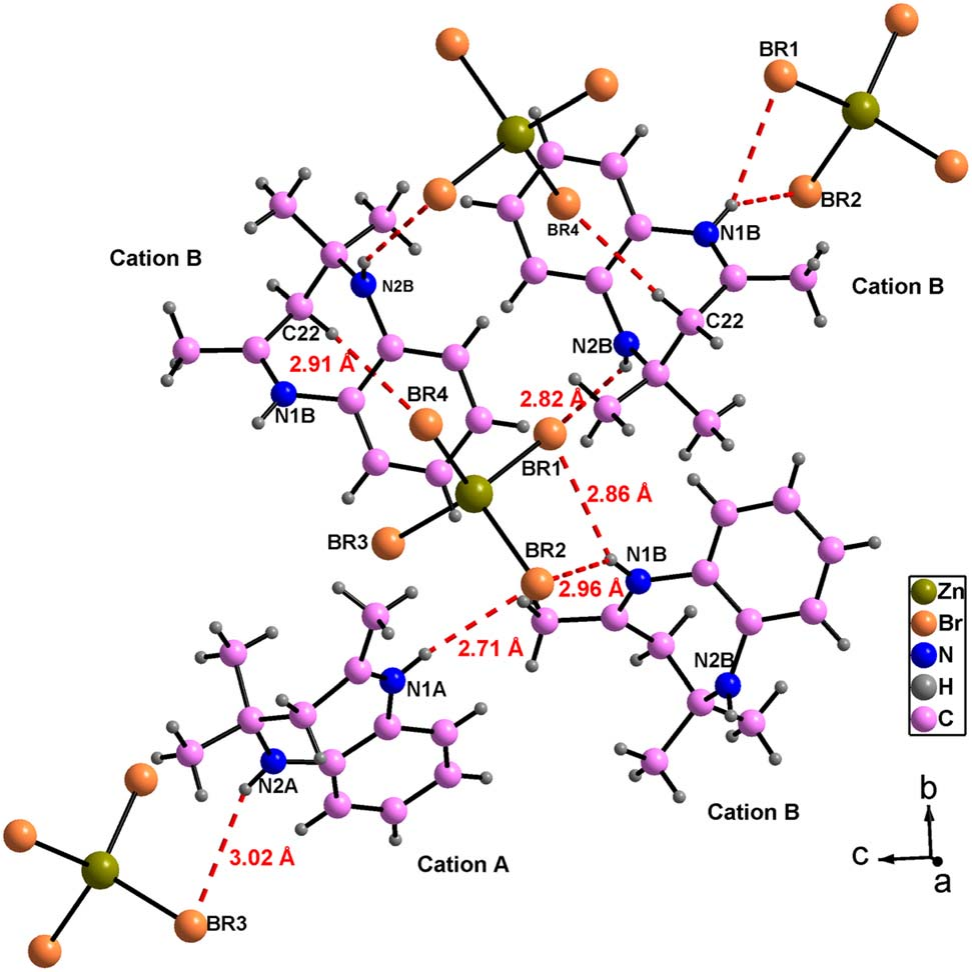
Hydrogen bonding around [ZnBr_4_]^2−^ anions.

The atomic arrangement of the studied compound is built of alternated organic and inorganic layers (Fig. 2). The crystal packing is stabilized by cation-to-anion N–H⋯Br and C–H⋯Br hydrogen bonds (four simple and one bifurcated) leading to a three-dimensional network. Hydrogen bonding parameters are listed in Table S3.† In this structure each [ZnBr_4_]^2−^ anions is surrounded by four organic (C_12_H_17_N_2_)^+^ cations (one cation A and three cations B (Fig. 3),). In addition, no face-to-face π–π interactions exist since the distance between centroid phenyl rings is equal to 7 Å.

### 3.2. Electrical impedance spectroscopy

Impedance spectroscopy is the most important technique to study the dynamics of mobile and bound charges in the bulk and interfacial areas of the materials. Generally, help in identifying the grain, grain boundary and electrode interface contributions in the polarization mechanism.^23,24^

**Fig. 4:**
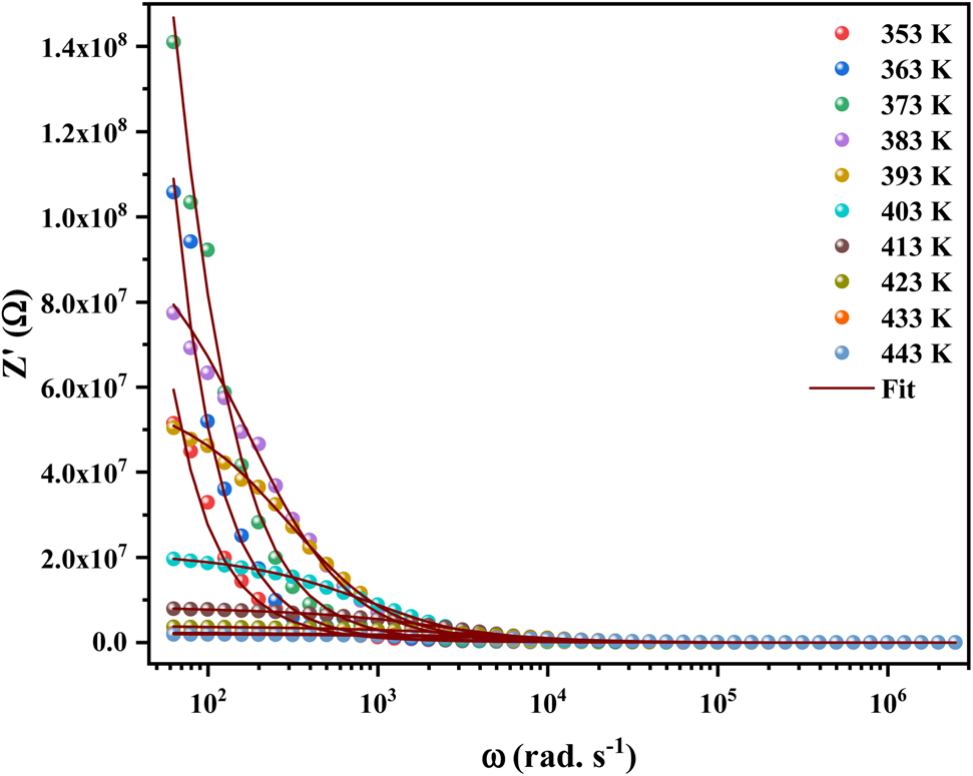
Variation of the real part (*Z*′) of the impedance of [C_12_H_17_N_2_]_2_ZnBr_4_ compound as a function of angular frequency for different temperatures.

Fig. 4 shows the variation of real part of the impedance (*Z*′) with frequency at different temperatures. At low frequencies, the magnitude of *Z*′ increases with the temperature at range from 353 K to 373 K. Then it is observed that beyond 373 K, the value of *Z*′ decreases on increasing temperature, which can be explained by the reduction of trapped charge density and a thermal activation of their mobility.^25^ Besides, the values of *Z*′ merge at high frequencies (>10^4^ Hz). This can be understood by the fact that charge carriers acquired sufficient energy to overcome the potential barrier.^26^

**Fig. 5:**
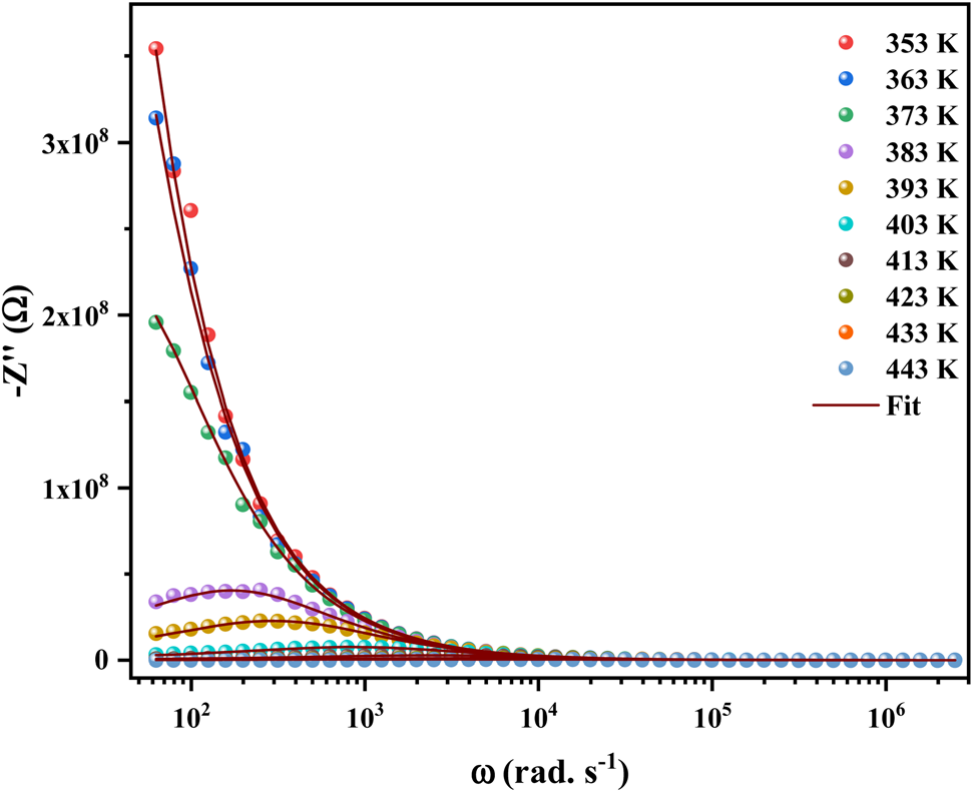
Variation of imaginary part (*Z*′′) with angular frequency in the measured temperature range.

Fig. 5 shows the temperature-dependent variations of the imaginary part (−*Z*′′) as a function of frequency over the temperature range of 353 K to 443 K. The peaks appearing in the plots of frequency *versus Z*′′_max_ were found to shift towards the side of higher frequencies. Furthermore, the broadening of peaks and decreasing value of *Z*′′_max_ with increasing temperature, indicating the fact that multiple relaxation processes are occurring simultaneously.^27^

The Nyquist plot between the real *Z*′ and imaginary (−*Z*′′) part of impedance is used to analyze the conduction mechanism in any sample. These graphs usually appear in the form of semicircles and each semicircle is a manifestation of unique relaxation process.^28^

**Fig. 6:**
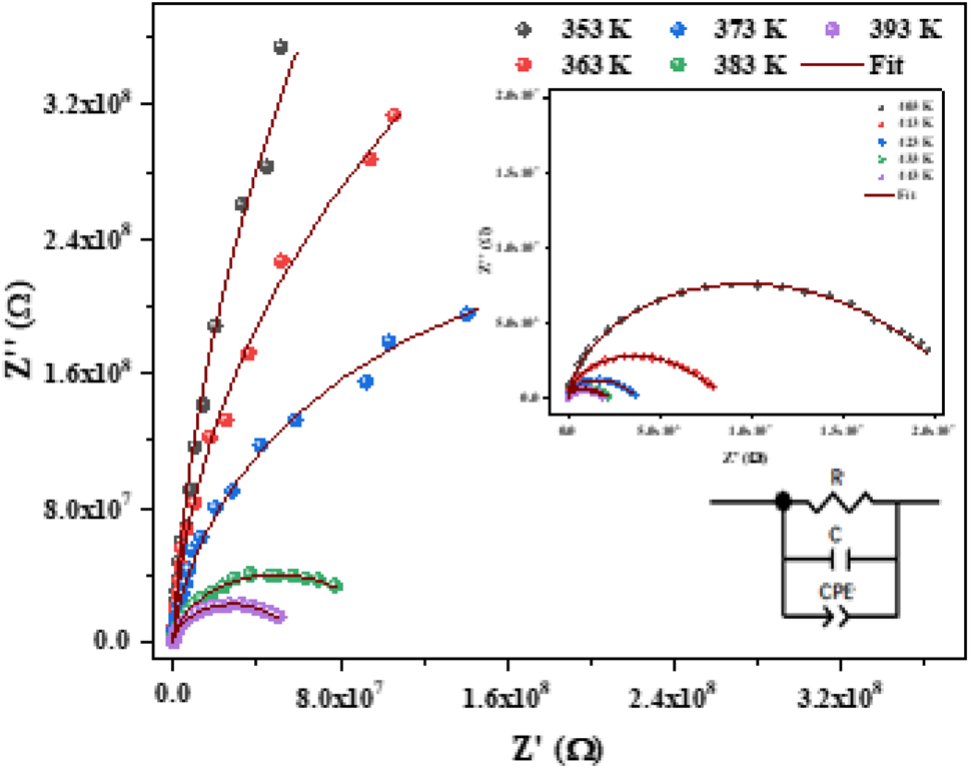
Nyquist plot fitted with an equivalent circuit for [C_12_H_17_N_2_]_2_ZnBr_4_ compound at different temperatures.

Fig. 6 shows the imaginary part of the impedance (−*Z*′′) *versus* the real part (*Z*′) over a wide range of frequencies and at different temperatures. The colored symbols demonstrate the actual experimental data, whereas the solid-red lines represent the fit administered by Zview software.^29^ This spectrum is characterized by the appearance of semicircular arc centered below the real axis which is temperature dependent. Depression of semicircle is originated from the presence of distribution of relaxation times. The radius of semicircle decreases with increasing temperature due to increase in conductivity of the material. In reality, the non-Debye type of relaxation is obtained which obeys Cole–Cole model.^30^ In order to analyze these spectra and to extract the different electrical parameters, an equivalent circuit model was proposed from the fit of experimental data to investigate the relationship between microstructure and electrical properties of sample. The best fit using Zview software was obtained using an equivalent circuit formed by a parallel combination of bulk resistance *R* capacitance C, and fractal capacitance CPE as depicted in the inset of Fig. 6. The constant phase element (CPE) is evaluated from the formula:1
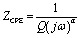
where, *Q* is the capacitance value of the CPE impedance and (0 < *α* < 1) relates to the deviation degree with respect to the pure capacitor value.

The theoretical values of the real (*Z*′) and imaginary (−*Z*′′) parts of thecomplex impedance, derived from the equivalent circuit, were deduced using the following expressions:2
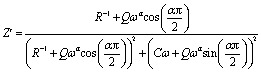
3
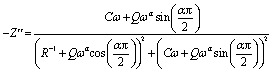


The parameters *R*, *C*, *α* and CPE obtained from the fitting results were evaluated and listed in Table 2. An overview in this table, the bulk resistance *R* decreases with increasing temperature, this behavior is related to increasing the mobility of charge carriers.^31^

**Table 2:** The extract parameters for the circuit elements

*T* (K)	*R* (Ω)	*C* (10^−11^ F)	*Q* (10^−11^ F)	*α*
353	2.83 × 10^9^	1.56	3.34	0.960
363	1.19 × 10^9^	2.09	3.07	0.943
373	5.06 × 10^8^	3.27	4.93	0.785
383	1.06 × 10^8^	3.46	20	0.621
393	6.18 × 10^7^	3.47	30.7	0.588
403	2.23 × 10^7^	3.49	84	0.513
413	8.76 × 10^6^	3.50	205	0.461
423	4.04 × 10^6^	3.50	434	0.426
433	2.45 × 10^6^	3.47	755	0.414
443	2.15 × 10^6^	3.44	960	0.412

**Fig. 7:**
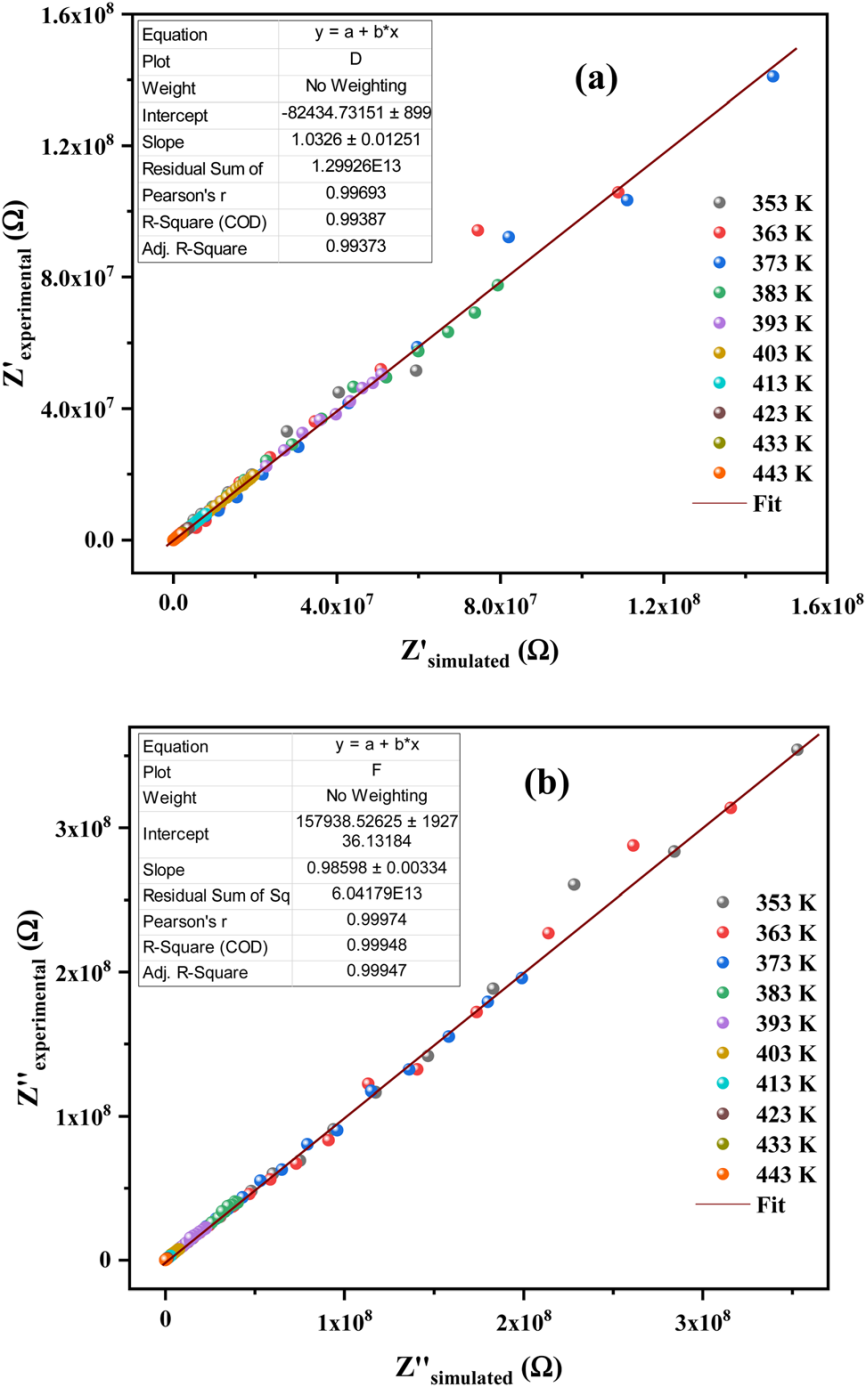
(a) and (b) Plots of measured values *versus* simulated values of the real and imaginary parts of the impedance.

The justification of the choice of the equivalent circuit is confirmed by the variations of the experimental values of (*Z*′) and (−*Z*′′) at various temperatures *versus* the calculated ones using the parameters of the equivalent circuit model (Fig. 7a and b). From this figure, it is evident that the slope obtained from a linear fit of the data points at each temperature is nearly equal to the unity. This behavior reveals that the adopted equivalent circuit describes well the electric properties of the investigated compound.

The electrical conductivity can be a well-established process for describing the hopping dynamics of the frees carriers. The obtained values of bulk resistance (*R*), corresponding to the grain, are used to determine the electrical conductivity sg as follows:4
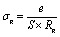
where *e* is sample thickness, *S* is the area of the pallet, *R*_g_ is grain resistance.

**Fig. 8:**
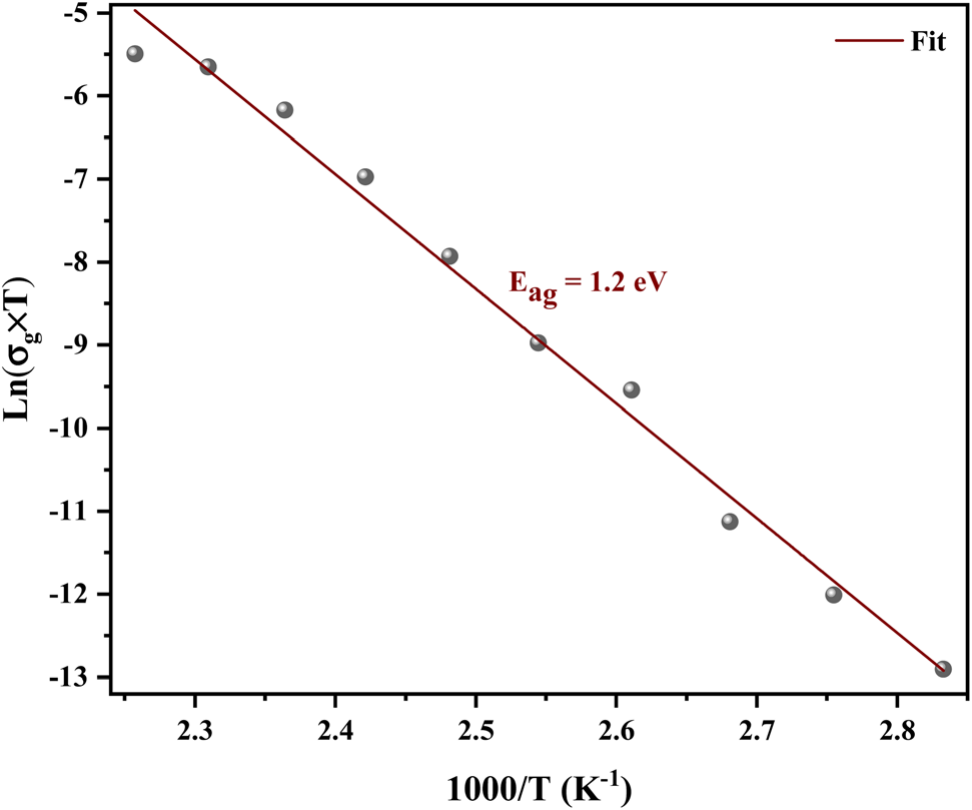
Arrhenius plots for the bulk conductivity of the [C_12_H_17_N_2_]_2_ZnBr_4_ sample.

Fig. 8 shows the variation of the conductivity *σ*_g_ for grains. The linearity of ln(*σ*_g_) *versus* 1000/*T* justifies that the title compound does not have a phase transition in the temperature range studied. We note that the conductivity increases with increase in temperature, which indicates the semiconductor behavior.^32^ The linear region is fitted with the Arrhenius equation:5
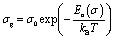
where *σ*_g_ is electrical conductivity, *σ*_0_ is the pre-exponential factor, *k*_B_ is the Boltzmann constant and *E*_a_ is the activation energy. The value of activation energy *E*_a_ can be determined from the linear fit is equal to: *E*_a_ = 1.2 eV. This activation energy *E*_a_ is similar to the values obtained previously.^32–34^

### 3.3. *ac* conductivity analysis

*ac* conductivity analysis helps to identify the type of transport for the charge carriers, which is responsible for the conduction process and its response as a function of frequency and temperature.^35^

**Fig. 9:**
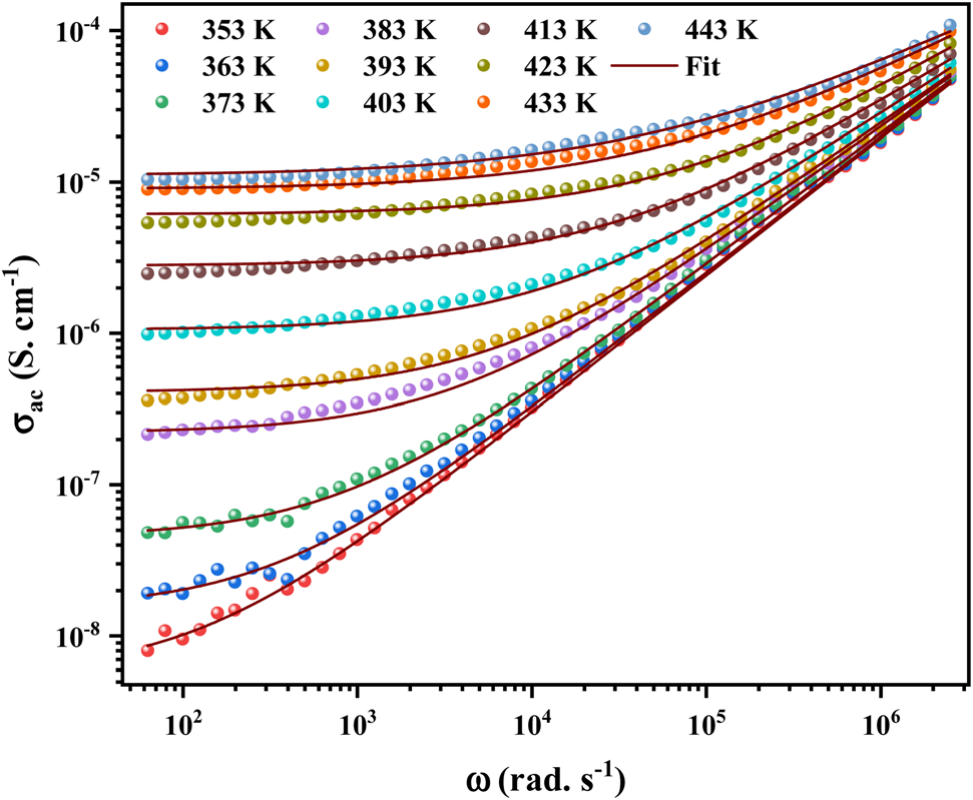
Variation of the total conductivity as a function of angular frequency at different temperatures for [C_12_H_17_N_2_]_2_ZnBr_4_ compound. Red solid lines represent the fitting to the experimental data using the universal Jonscher power law.

The angular frequency dependence of the *ac* conductivity at several temperatures for [C_12_H_17_N_2_]_2_ZnBr_4_ compound is shown in Fig. 9. The conductivity spectrum can be visually defined for two distinct regions. In the first region, a plateau is remarked at a low frequency up to a specific value known as the hopping frequency *ω*_h_ = 10^5^ rad s^−1^, which indicates a frequency-independent conductivity. The conductivity increasing with temperature, this implies a semiconductor behavior for the prepared sample.^36^ For the second region, the conductivity increases with increasing frequency. Moreover, to identify the suitable mechanism *ac* conductivity, an analysis of the frequency and conductivity (*σ*_*ac*_) data are fitted by the Jonscher power law:^37^

6 *σ*_*ac*_ (*ω*) = *σ*_*dc*_ + *Aω*^*s*^

where *σ*_*dc*_ is the *dc* conductivity, *s* represents the degree of interaction between mobile ions and the environments surrounding them and *A* is a constant. The *A* and *s* factors vary according to the temperature and the nature of the sample.

To define the predominant conduction mechanism of the *ac* conductivity of [C_12_H_17_N_2_]_2_ZnBr_4_ compound, several theoretical models correlating the conduction mechanism with the exponent *s*(*T*) behavior were used to determine this objective. We have fitted the *ac* conductivity data by using eqn (4) and the best fits can be obtained by consecutively varying *A* and *s* parameter.

**Fig. 10:**
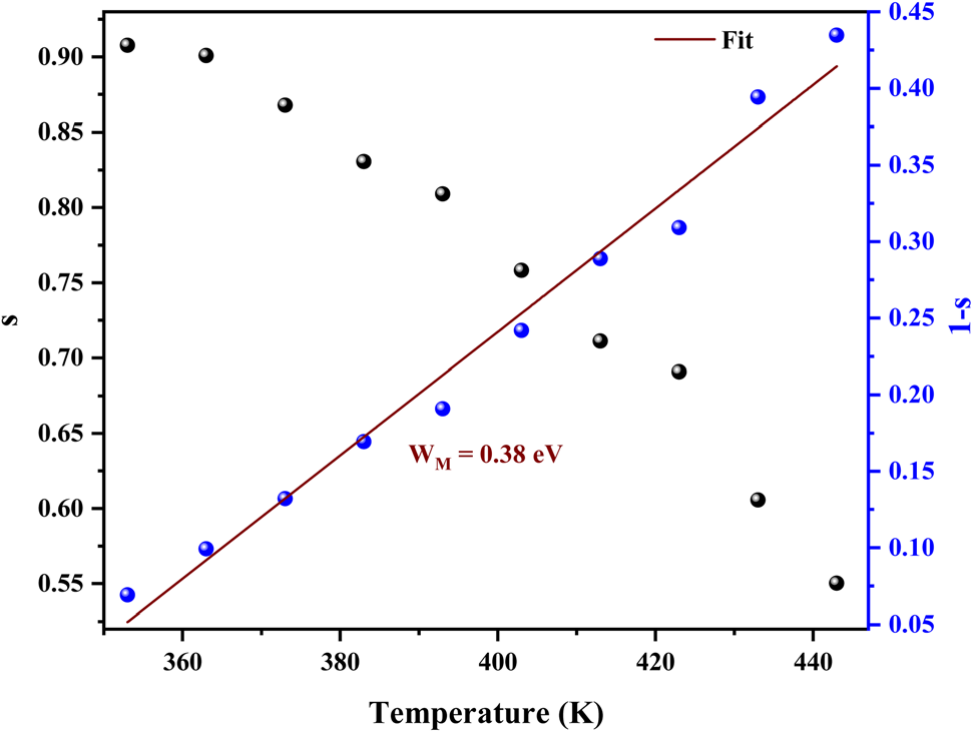
Temperature dependence of the frequency exponent s and 1 s.

From Fig. 10 it is possible to determine the conduction process in the prepared sample. Thus, *s* tends to decrease with increasing temperature. This decreasing trend behavior of *s* with temperature suggests that the conduction mechanism in the compound can be explained by the Correlated Barrier Hopping (CBH) model in the sample.^38^

In the CBH model, the charge carriers can move from one location to another by performing a hopping over a potential barrier.^39^ The frequency exponent *s* is represented by following equation:7
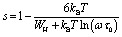
where *τ*_0_ is a characteristic relaxation time *τ*_0_ = 10^−13^. *s* and *W*_H_ represents the energy needed to move an electron from one location to another one. For the large values of *W*_H_/*k*_B_*T* is considered, eqn (7) reduces to eqn (8):^40^8
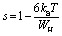


The average value of the barrier energy *W*_H_*average* was calculated as 0.31 eV by the slope of the line given in Fig. 7. This value is approximately a quarter of the activation energies 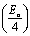 which indicates that the single polaron hopping is the dominating conduction mechanism in [C_12_H_17_N_2_]_2_ZnBr_4_.^41^

In this formalism, the alternating conductivity *ac* is given by:^42–44^9
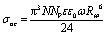
where *NN*_P_ is proportional to the square of the concentration of states, *ε* is the dielectric constant of the material and *R*_*ω*_ is the hopping distance for conduction and is given by the relation:10
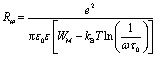


*NN*_P_ is given by: *NN*_P_ = *N*_T_^2^ (for bipolaron hopping), where *N*_T_ is the number of states density. *NN*_P_ = *N*_T_^2^ e^(−*U*_eff_/2*k*_B_*T*)^ (for single polaron hopping).

**Fig. 11:**
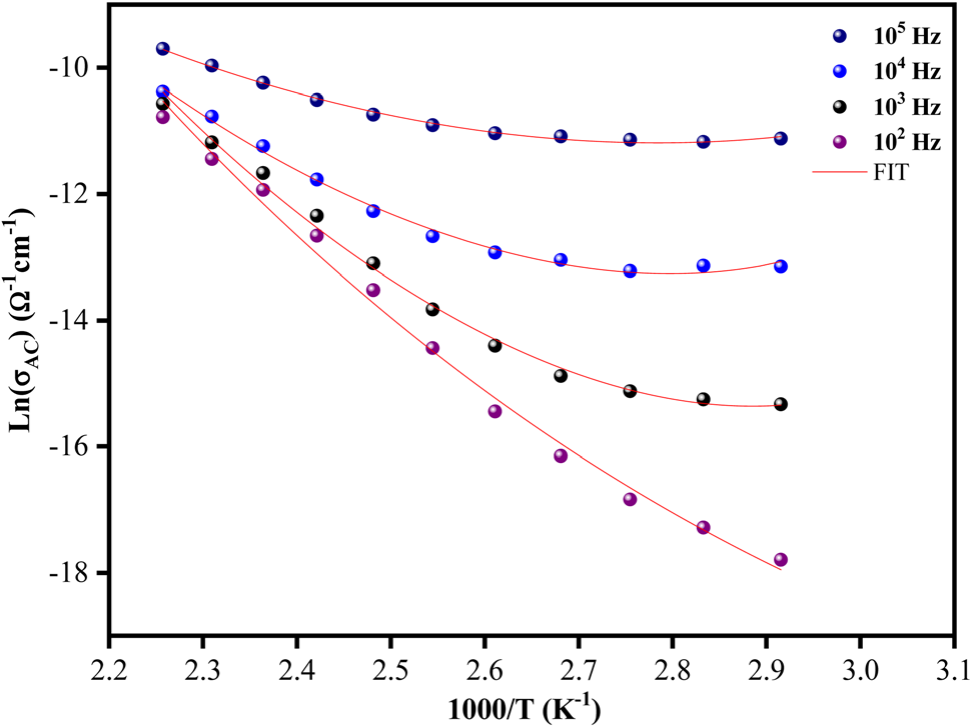
Evolution of the ln(*σ*_*ac*_) *versus* 1000/*T* for the [C_12_H_17_N_2_]_2_ZnBr_4_ compound.

The temperature dependence of the *ac* conductivity for [C_12_H_17_N_2_]_2_ZnBr_4_ at selected frequencies is reported in Fig. 11. Clearly this plots shows that the theoretical calculations fitted by eqn (9) are good with the experimental data. The calculated fitting parameters are listed in Table 3. It can be noted that the values of the density of states *N*(*E*_F_) as a function of frequency are reasonable for localized states.^45^ Due to the strong electron–phonon interaction, the effective Hubbard intrasite correlation energy *U*_eff_ is found to be negative.^46^

**Table 3:** *ac* conductivity parameters used for CBH model fitting for the [C_12_H_17_N_2_]_2_ZnBr_4_ hybrid material at various frequencies

*F* (Hz)	*N*_EF_ (eV^−1^ m^−1^)	*U*_eff_
10^2^	6.8018 × 10^13^	−0.08364
10^3^	4.4729 × 10^14^	−0.08933
10^4^	5.3397 × 10^15^	−0.0692
10^5^	1.1309 × 10^16^	−0.04252

### 3.4. Optical properties

#### 3.4.1. UV-visible absorption spectra analysis

**Fig. 12:**
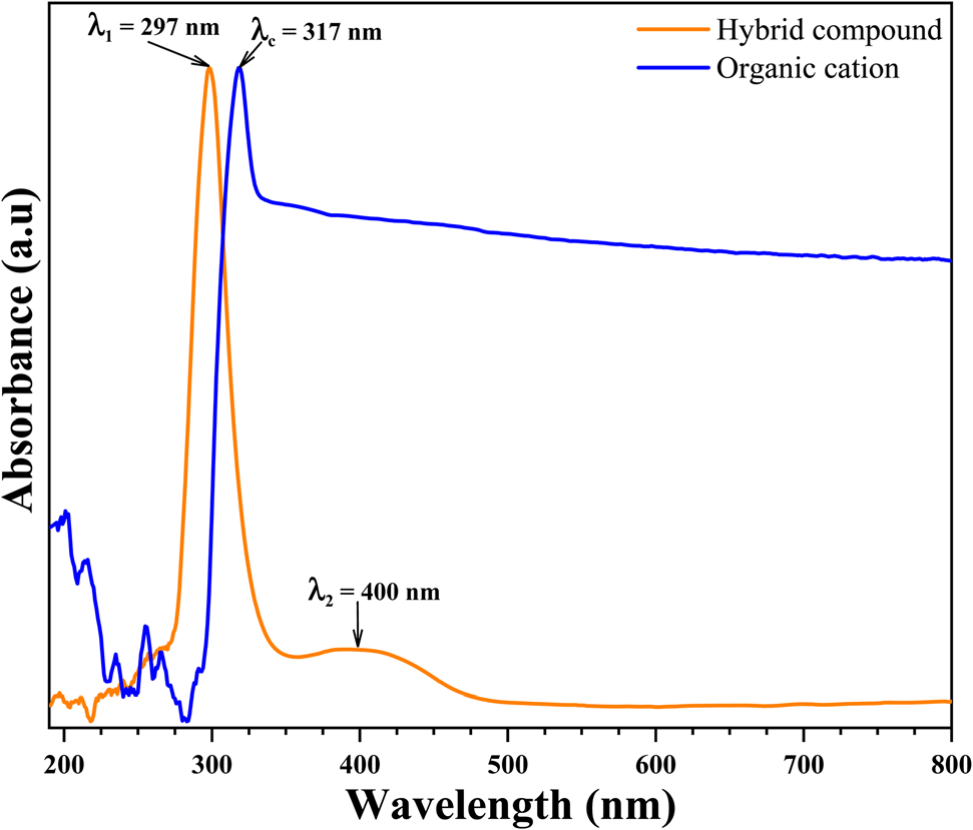
UV-visible absorption spectra of organic cation (blue line) and organic-inorganic hybrid compound (orange line).

UV radiation is therefore an effective tool for the identification and characterization of the structure and absorption of the materials. The absence of absorption bands in the visible region of the grown compound attests to the suitability of the grown materials for photonic and optical applications.^47^ The UV-visible absorption spectra of organic cation and cluster study of the spin-coated film was recorded in a range of 200–800 nm (Fig. 12).

As can be seen Fig. 12, the absorption spectrum of organic cation shows one strong distinguished peak centered at *λ*_*c*_ = 317 nm. This strong absorption peak in the UV region have been attributed to π → π* transitions in the organic cation ring. This peak has been shifted to the strong distinguished peak centered at *λ*_1_ = 297 nm appeared in the absorption spectrum of hybrid compound. This shift caused by the new environment (inorganic anion) characterized by the charge transferred from organic cation the inorganic anion. Another medium band centered at *λ*_2_ = 400 nm is also showed in the absorption spectrum of hybrid compound in visible region. This absorption band have been attributed to metal charge transferred in inorganic anion from metal atom (Zn) to halogen atom (Br).

#### 3.4.2. Band gap analysis

The determination of the optical bandgap *E*_g_ was based on the Tauc formula. Since the band gap energy (*E*_g_) controls the optical and electrical properties of materials, it is essential to know and discuss this quantity. The study of the electronic structure and the type of transition of the electrons shows us the dependence of the coefficient of optical absorption with the energy of the photons. Using the absorption data, the optical absorption coefficient “*α*” can be deduced by applying the Beer–Lambert's law:^48^11
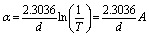
where, *A* is the absorbance and *d* is the thickness of the thin layer.

The absorption coefficient is given by the following Pankove's relation:^49^12
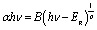


The coefficient *p* = 2 for allowed direct transitions and *p* = 0.5 for indirect allowed transitions. *B* is the probability parameter for the transition and *E*_g_ the optical bandgap energy.

The *E*_g_ value corresponding to direct band gap transitions can be calculated *via* the (*αhν*)^2^*versus hν*, using the formula:

13 (*αhν*)^2^ = *B*(*hν* − *E*_g_)

The *E*_g_ value corresponding to indirect band gap transitions can be considered *via* the (*αhν*)^0.5^*versus hν*, using the formula:

14 (*αhν*)^0.5^ = *B*(*hν* − *E*_g_)

**Fig. 13:**
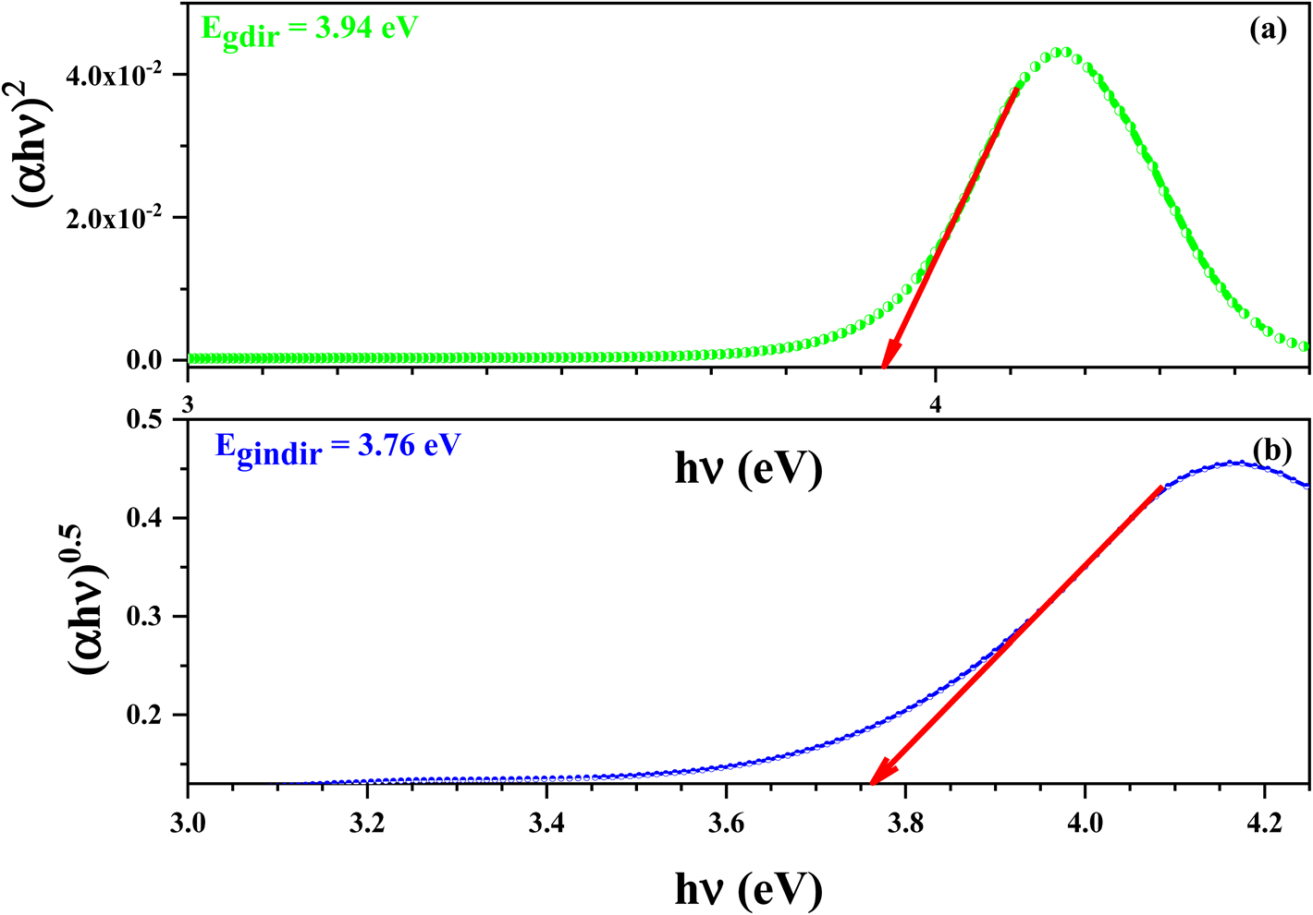
Variations of (*αhν*)^2^ (a) and (*αhν*)^0.5^ (b) *versus hν* for the [C_12_H_17_N_2_]_2_ZnBr_4_ compound.

Fig. 13 demonstrate the variation of (*αhν*)^2^ (a) and (*αhν*)^0.5^ (b) *versus hν* of this compound. The values of *E*_gd_ and *E*_gind_ were estimated from the intersection of the extrapolated linear part of the (*αhν*)^2^ and (*αhν*)^0.5^ curves with energy axis.

The values of *E*_gd_ and *E*_gind_ were estimated from the intersection of the extrapolated linear part of the (*αhν*)^2^ and (*αhν*)^0.5^ curves with energy axis. The optical absorption measurement near the fundamental absorption edge is a standard method for estimation of the band gap energy. The direct band gap of the 3.94 eV results in an energy gap of 3.76 eV.

## 4. Conclusion

In this work, a new [C_12_H_17_N_2_]_2_ZnBr_4_ sample, with a centrosymmetric *P*2_1_/*n* space group, was prepared and its optical, electrical and *ac* conductivity are investigated as a function of temperature and frequency. The asymmetric unit is constituted by one tetabromozincate [ZnBr_4_]^2−^ anion and two protonated [C_12_H_17_N_2_]^+^ cations.

The energy of the optical gap was found to be approximately 3.94 eV for a direct transition and 3.76 eV for an indirect one. This leads us to conclude that the [C_12_H_17_N_2_]_2_ZnBr_4_ compound is a good candidate for the application of semiconductors.

The Nyquist plots were satisfactorily fitted with the one-cell circuit model R/C/CPE, with the exception of adding a CPE element in series from the temperature of phase transition. The temperature dependency of *Z*′ with an inverse variation suggests a semiconducting behavior. The *ac* conductivity, over the studied temperature and frequency range, is described by Jonscher's power law. The thermal behavior of the extracted exponent *s* confirmed that the CBH model is the appropriate model for this compound.

## Conflicts of interest

The authors declare that they have no known competing financial interests or personal relationships that could have appeared to influence the work reported in this paper.

## Supplementary Material

RA-013-D3RA00561E-s001

RA-013-D3RA00561E-s002
