# Supplementary material for: Investigation of structural, optical and electrical conductivity of a new organic inorganic bromide: [C12H17N2]2ZnBr4
Source: RSC Adv. 2023 Mar 10;13(12):8034–42. doi: 10.1039/d3ra00561e (PMC9999369; doi:10.1039/d3ra00561e)
Supplement: RA-013-D3RA00561E-s001 [file RA-013-D3RA00561E-s001.pdf]

Electronic Supplementary Material (ESI) for RSC Advances.
This journal is © The Royal Society of Chemistry 2023

Table S1: Bond lengths (Å) and angles (°) of the $[ZnBr_4]^{2-}$ tetrahedron

**$[ZnBr_4]^{2-}$ anions**			
Zn—Br4	2.3638 (11)	Br4—Zn—Br3	108.27 (4)
Zn—Br3	2.3972 (10)	Br4—Zn—Br1	110.69 (4)
Zn—Br1	2.4113 (11)	Br3—Zn—Br1	112.50 (4)
Zn—Br2	2.4464 (10)	Br4—Zn—Br2	113.89 (4)
		Br3—Zn—Br2	106.12 (4)
		Br1—Zn—Br2	105.35 (4)

Table S2: Bond lengths (Å) and angles (°) of the organic cations of $[C_{12}H_{17}N_2]_2[ZnBr_4]$

**Organic Cations**			
N1B—C23	1.284(8)	C5—C4	1.359(10)
N1B—C13	1.421(9)	C3—C4	1.372(10)
C1—C2	1.382(9)	C13—C18	1.358(10)
C1—C6	1.393(9)	C13—C14	1.371(10)
C11—C12	1.464(9)	C19—N2B	1.454(9)
C11—C10	1.489(10)	C23—C24	1.440(10)
C7—N2A	1.430(9)	C14—C15	1.341(10)
C7—C9	1.503(10)	C18—C17	1.385(11)
C7—C10	1.529(9)	C17—C16	1.375(11)
C7—C8	1.539(10)	C16—C15	1.378(11)
C2—C3	1.384(9)	C22—C19	1.510(9)
C6—N2A	1.354(9)	C22—C23	1.489(9)
C6—C5	1.407(9)		
C11—N1A—C1	133.3(6)	C18—C13—C14	120.0(7)
C23—N1B—C13	127.6(7)	C18—C13—N1B	121.9(6)
C2—C1—C6	120.9(6)	C14—C13—N1B	118.0(7)
C2—C1—N1A	113.6(6)	N2B—C19—C20	111.2(8)
C6—C1—N1A	125.5(6)	N2B—C19—C22	109.7(6)
N1A—C11—C12	119.4(7)	C20—C19—C22	112.6(7)
N1A—C11—C10	119.5(6)	N2B—C19—C21	105.2(7)
C12—C11—C10	121.1(7)	C20—C19—C21	108.5(9)
N2A—C7—C9	110.9(7)	C22—C19—C21	109.4(7)
N2A—C7—C10	110.3(6)	C11—C10—C7	114.3(6)
C9—C7—C10	110.6(6)	C5—C4—C3	120.8(7)
N2A—C7—C8	106.6(6)	C18—N2B—C19	122.8(7)
C9—C7—C8	109.7(7)	N1B—C23—C24	119.8(7)
C10—C7—C8	108.7(6)	N1B—C23—C22	117.6(7)
C1—C2—C3	121.3(7)	C24—C23—C22	121.7(7)
N2A—C6—C1	127.1(6)	C15—C14—C13	122.4(8)
N2A—C6—C5	116.7(7)	C13—C18—C17	119.0(7)
C1—C6—C5	116.2(7)	C13—C18—N2B	119.7(7)
C6—N2A—C7	130.0(6)	C17—C18—N2B	120.6(8)
C23—C22—C19	113.8(6)	C16—C17—C18	119.6(8)
C4—C5—C6	122.4(7)	C17—C16—C15	121.0(8)
C4—C3—C2	118.3(7)	C14—C15—C16	118.0(8)

Table S3: Hydrogen bonds geometry of of $[C_{12}H_{17}N_2]_2[ZnBr_4]$

D—H···*A*	*D*—H	H···*A*	*D*···*A*	*D*—H···*A*
N1A—H1A···Br2^{i}	0.86	2.71	3.566(5)	171
N1B—H1B···Br1^{ii}	0.86	2.86	3.578(6)	142
N1B—H1B···Br2^{ii}	0.86	2.96	3.626(6)	135
N2A—H2A···Br3^{iii}	0.86	3.02	3.806(7)	153
N2B—H2B···Br1	0.86	2.82	3.576(6)	147
C22—H22A···Br4^{iv}	0.97	2.91	3.876(7)	174

Symmetry codes: **(i)** *x*, *y*+1, *z*; **(ii)** −*x*+3/2, *y*+1/2, −*z*+3/2; **(iii)** −*x*+3/2, *y*+1/2, −*z*+1/2; **(iv)** −*x*+1, −*y*+1, −*z*+1.
